# Cork By-Products as Bioactive Ingredients: From Waste Valorization to Pharmaceutical Prototypes

**DOI:** 10.3390/molecules31010095

**Published:** 2025-12-25

**Authors:** Nuno Miguel Silva, Ana Colette Maurício, Ruben Fernandes, Ana Novo Barros

**Affiliations:** 1 Unidade Local de Saúde Santo Antonio (ULSSA), Instituto de Ciências Biomédicas de Abel Salazar (ICBAS), Universidade do Porto (UP), 4099-002 Porto, Portugal; nunosilva.farmacia@ulssa.min-saude.pt; 2 Centro de Estudos de Ciência Animal (CECA), Instituto de Ciências, Tecnologias e Agroambiente da Universidade do Porto (ICETA), 4051-401 Porto, Portugal; acmauricio@icbas.up.pt; 3 Departamento de Clínicas Veterinárias, Instituto de Ciências Biomédicas de Abel Salazar (ICBAS), Universidade do Porto (UP), 4099-002 Porto, Portugal; 4 Associate Laboratory for Animal and Veterinary Science (AL4AnimalS), 1300-477 Lisboa, Portugal; 5 RISE-Health, Escola de Medicina e Ciências Biomédicas (EMCB), Universidade Fernando Pessoa (UFP), Fundação Ensino e Cultura Fernando Pessoa (FFP), Avenida Fernando Pessoa, 150, 4420-096 Gondomar, Portugal; ruben.fernandes@ufp.edu.pt; 6 Centre for the Research and Technology of Agroenvironmental and Biological Sciences, CITAB, Inov4Agro, Universidadede Trás-os-Montes e Alto Douro, UTAD, Quinta de Prados, 5000-801 Vila Real, Portugal

**Keywords:** cork by-products, bioactive compounds, antioxidant activity, anti-inflammatory activity, anti-aging, dermocosmetics, pharmaceutical applications, sustainability, circular, bioeconomy

## Abstract

The pharmaceutical sector has evolved toward innovation-driven and sustainability-oriented development, driven by increasing regulatory pressure and global health challenges. In this context, cork (*Quercus suber* L.) has emerged as a promising bio-based resource due to its renewable nature, near-zero-waste processing chain, and growing evidence of biological activity. Cork by-products are rich in phenolic compounds, triterpenes, lignin derivatives, and other secondary metabolites exhibiting antioxidant, anti-inflammatory, and anti-aging properties, with relevance for pharmaceutical and dermocosmetic applications. These bioactivities are associated with the modulation of oxidative stress, inhibition of pro-inflammatory signaling pathways, and support of skin barrier function. This review provides an updated and focused overview of the chemical composition, bioactive potential, and valorization pathways of cork by-products, with particular emphasis on their translation into pharmaceutical and dermocosmetic formulations. Key challenges related to extraction standardization, bioavailability, safety, and clinical validation are critically discussed, highlighting future directions for the sustainable development of cork-derived bioactive ingredients within circular bioeconomy frameworks.

## 1. Introduction

**Figure 1:**
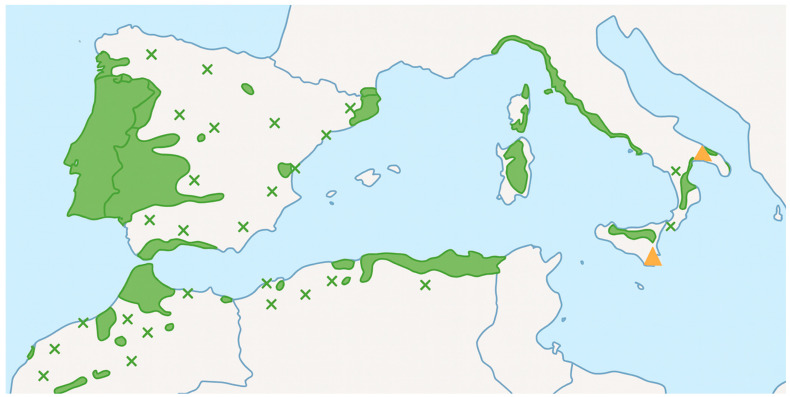
Geographical distribution of cork oak (*Quercus suber* L.) in the Mediterranean basin and along the Atlantic coast of mainland Portugal. Green areas indicate native distribution; crosses represent native but isolated populations; triangles indicate introduced and naturalized stands. Adapted from [4].

Cork (*Quercus suber* L.) is a renewable, biodegradable, and versatile material predominantly distributed across the western Mediterranean region, where its harvesting has held socio-economic and ecological relevance for centuries [1,2]. Traditionally, cork has been used in wine stoppers, construction materials, and thermal or acoustic insulation. However, a significant portion of the cork processed globally—particularly cork powder, granulates, and lower-grade bark—remains underexploited despite being generated in large quantities during industrial transformation (Figure 1) [1,3].

These by-products are known to be rich in bioactive molecules such as phenolic acids, flavonoids, ellagitannins, and triterpenes [5,6]. Several studies have highlighted the biological relevance of these compounds, reporting antioxidant, anti-inflammatory, and anti-aging activities that support their potential application in health-related formulations [7,8]. Triterpenes such as betulinic acid, friedelin, and lupeol—characteristic of cork’s chemical profile—also stand out for their pharmacological interest [1,9].

Progress in extraction techniques and analytical characterization has further reinforced the value of cork residues as a source of high-added-value natural bioactives [7,10]. Nonetheless, most existing reviews focus either on the general composition of cork or on its applications as a sustainable material, leaving a notable gap regarding the transition from industrial by-products to ingredients with pharmaceutical relevance.

**Figure 2:**
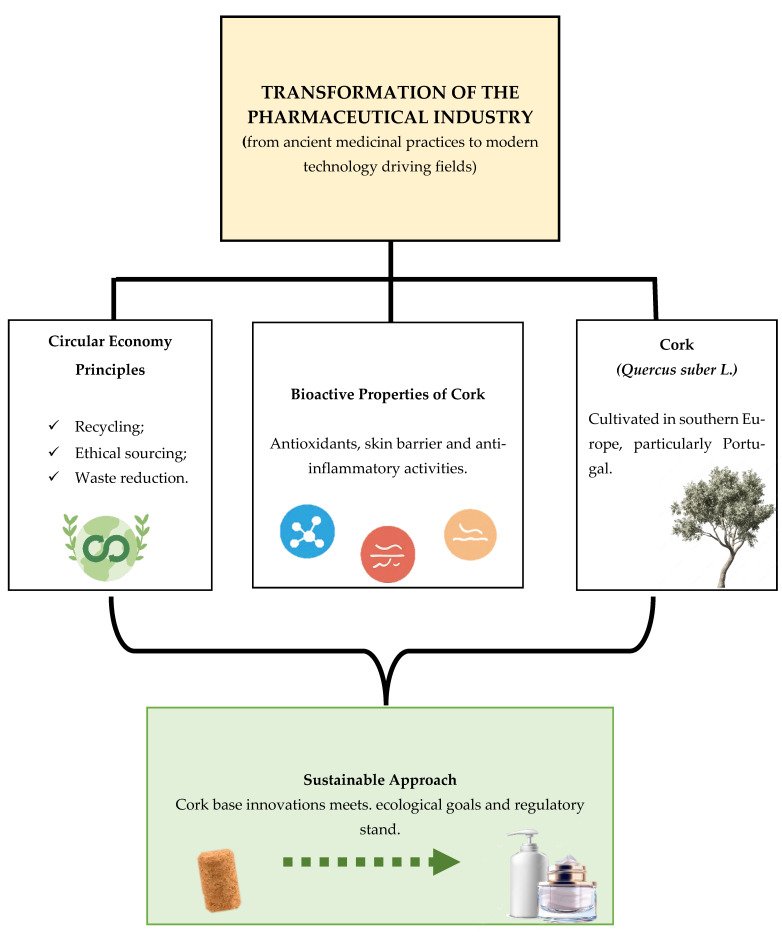
Conceptual framework illustrating the transition of the pharmaceutical industry toward sustainable, bio-based innovation, highlighting cork (*Quercus suber* L.) as a renewable source of bioactive compounds.

This review aims to address that gap by examining the chemical composition, bioactive potential, and valorization pathways of cork by-products with a particular focus on their applicability in pharmaceutical and dermocosmetic prototypes. By integrating concepts from green extraction, circular bioeconomy, and early drug formulation, this work offers a fresh perspective on the sustainable and functional exploitation of cork residues, emphasizing both their industrial value and their therapeutic promise (Figure 2).

## 2. Types and Chemical Characterization of Cork Residues

Cork processing generates several types of by-products that have traditionally been treated as low-value materials but are now recognized as promising sources of bioactive molecules. The most common residues include cork powder, produced during sanding and shaping operations; cork granulates, obtained from lower-quality or defective cork unsuitable for stopper production; and bark fragments resulting from the initial debarking and preparation stages [1,3]. Although these materials historically had limited industrial use, recent interest in sustainable material streams has highlighted their potential as renewable raw materials for pharmaceutical, dermocosmetic, and nutraceutical applications.

Chemically, cork residues display a highly heterogeneous and multifunctional composition. Their primary component is suberin, a hydrophobic and structurally complex polyester composed of long-chain fatty acids, glycerol, and aromatic domains. Suberin is responsible for cork’s characteristic elasticity, low permeability, and chemical resistance [5,6]. Another major constituent is lignin, a phenylpropanoid polymer that reinforces the cell wall structure and contributes to both mechanical strength and intrinsic antioxidant capacity [6].

In addition to these macromolecular components, cork residues contain polysaccharides—notably cellulose and hemicelluloses—which provide structural support and participate in interactions with extractable phenolics [5]. The outer bark and powder fractions also contain waxes and triterpenoids, including lupeol, friedelin, and betulinic acid, which enhance barrier properties and possess well-documented anti-inflammatory, wound-healing, and anti-aging activities [1,9].

**Figure 3:**
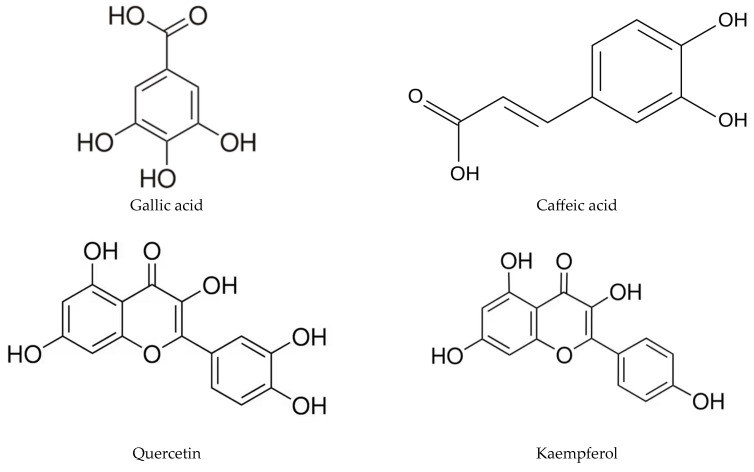

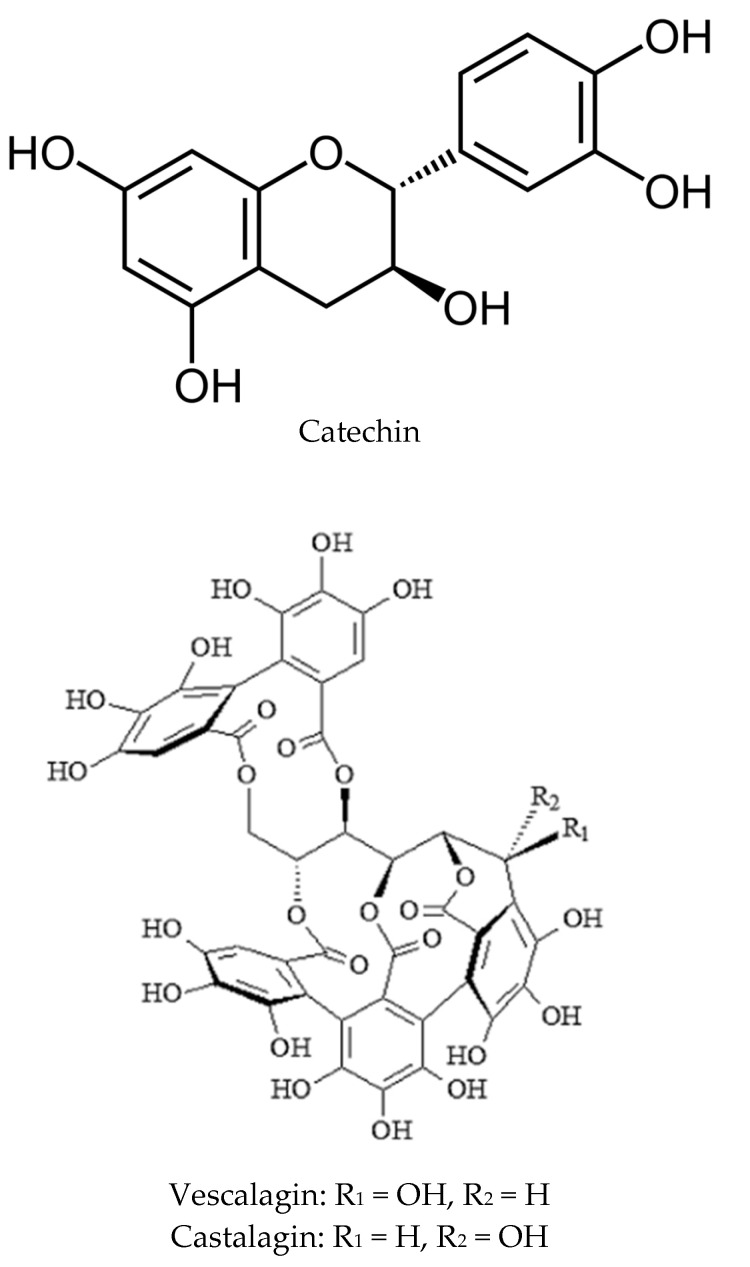
Representative bioactive compounds identified in cork by-products. Major phenolic acids (gallic acid, caffeic acid), flavonoids (quercetin, kaempferol, catechin), and ellagitannins (vescalagin, castalagin), commonly reported in cork residues and associated with antioxidant and anti-inflammatory activities. Adapted from [11].

Among the most pharmacologically relevant constituents are phenolic compounds, including gallic and caffeic acids, ellagitannins, catechins, quercetin, and kaempferol (Figure 3).

These have been repeatedly associated with strong antioxidant, anti-inflammatory, and enzyme-modulating effects [7,8]. Recent studies show that cork by-products often contain higher concentrations of phenolics than commercial cork planks, due to greater surface area and exposure during industrial processing [7,10].

It is also increasingly recognized that the chemical profile of cork residues is influenced by geographical origin, climatic conditions, soil characteristics, and genetic variability within Quercus suber populations [1,2]. These factors can affect both extraction yields and the bioactivity of the resulting fractions, highlighting the importance of standardizing raw materials for future pharmaceutical applications.

A detailed understanding of the compositional diversity of cork residues is essential for developing targeted extraction strategies and ensuring reproducible biological effects. By leveraging this chemical richness—traditionally overlooked due to the perception of cork by-products as waste—researchers can unlock new avenues for sustainable and high-value applications, particularly in the development of natural bioactive ingredients and early-stage pharmaceutical prototypes.

## 3. Extraction Strategies and Optimization for Bioactive Recovery

The efficient extraction of bioactive compounds from cork by-products is a critical step in transforming these materials into functional ingredients suitable for pharmaceutical applications. Over the past decade, advances in green extraction technologies have enabled the selective recovery of phenolic compounds, triterpenoids, and other bioactives while reducing solvent consumption and improving overall sustainability [7,10].

Traditional extraction methods, such as solid–liquid extraction using hydroethanolic solvents, remain widely employed due to their simplicity, reproducibility, and suitability for both polar and moderately non-polar compounds. Hydroethanolic mixtures (typically 50–80% ethanol) have consistently demonstrated high efficiency in recovering phenolic acids, flavonoids, and ellagitannins from cork residues [7]. However, these conventional techniques often require long extraction times and elevated temperatures, which may lead to partial degradation of thermolabile compounds.

To overcome these limitations, more sustainable and time-efficient approaches have gained prominence. Ultrasound-assisted extraction (UAE) enhances solvent penetration and mass transfer, enabling higher yields of phenolics in significantly shorter extraction times. UAE has been shown to improve the recovery of caffeic acid, gallic acid, and ellagitannins from cork powder while reducing energy inputs [10]. Similarly, microwave-assisted extraction (MAE) offers rapid heating and improved disruption of cell wall structures, facilitating the release of triterpenoids and other moderately hydrophobic compounds embedded within suberin-rich matrices.

Another promising approach is supercritical CO_2_ extraction, particularly valuable for recovering triterpenes such as friedelin, lupeol, and betulinic acid. This technique avoids organic solvents and enables the selective extraction of lipophilic molecules under controlled pressure and temperature conditions [9]. When combined with ethanol as a cosolvent, supercritical CO_2_ can broaden the extraction spectrum to include waxes and semi-polar constituents relevant to dermocosmetic and pharmaceutical formulations.

Beyond extraction efficiency, optimization studies have become increasingly important to ensure scalability and reproducibility. Parameters such as particle size, solvent polarity, extraction time, and temperature significantly influence yield and composition. For instance, fine cork powder typically provides higher extraction efficiency due to its increased surface area, while moderate heating (40–60 °C) improves the solubility of certain phenolics without inducing degradation [6]. Response surface methodologies and multivariate optimization tools have been applied to identify conditions that maximize antioxidant and anti-inflammatory activity, rather than simply maximizing mass yield.

Importantly, the choice of extraction technique directly impacts the bioactive profile and potential pharmaceutical applications of the resulting extracts. Hydroethanolic methods tend to favor antioxidant-rich phenolics suitable for anti-aging formulations, whereas supercritical extraction preferentially yields triterpenoids with anti-inflammatory and wound-healing properties [8,9]. This alignment between extraction selectivity and intended biological function is essential for the rational design of cork-derived pharmaceutical prototypes.

Overall, the progression from conventional to advanced green extraction strategies underscores the feasibility of valorizing cork residues through efficient and scalable recovery of bioactive compounds. Establishing optimized protocols not only supports sustainable use of industrial by-products but also provides a critical foundation for developing standardized, high-quality extracts suitable for subsequent formulation and preclinical evaluation.

## 4. Biological Activities and Mechanisms of Action

**Figure 4:**
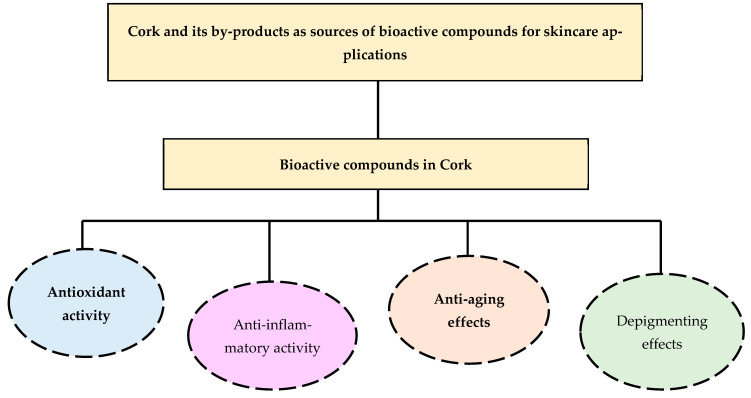
Main biological activities of cork-derived bioactive compounds relevant to topical and pharmaceutical applications.

Cork and its by-products are increasingly recognized as sustainable sources of bioactive compounds for topical pharmaceutical and cosmetic applications. Rich in phenolic acids, flavonoids, triterpenoids, and tannins, cork exhibits a wide spectrum of biological activities, including antioxidant, anti-inflammatory, anti-aging, radical-scavenging, and depigmenting effects [12]. These multifunctional properties render cork-derived extracts particularly attractive for developing natural, eco-friendly skincare solutions, satisfying the growing consumer demand for sustainable and multifunctional products (Figure 4).

Cork phenolics and triterpenoids exhibit antioxidant, anti-inflammatory, anti-aging, antimicrobial, and barrier-protective effects through modulation of oxidative stress and inflammatory pathways. Beyond their relevance for topical and dermocosmetic applications, several cork-derived constituents—particularly triterpenoids such as friedelin, betulinic acid, and lupeol, as well as phenolic acids and ellagitannins—have demonstrated broader pharmacological potential. Studies report antimicrobial, anti-inflammatory, hepatoprotective, neuroprotective, cardioprotective, and anticancer properties, expanding the therapeutic relevance of cork far beyond skin health. These activities are largely mediated through well-characterized molecular pathways, including NF-κB, Nrf2/ARE, MAPKs, COX/LOX inhibition, and modulation of oxidative and inflammatory cascades.

### 4.1. Antioxidant Activities

Ellagitannins, vanillin, and friedelin are among the most potent antioxidant constituents identified in cork, collectively forming a multifunctional protective system relevant for cosmetic and dermatological applications. Ellagitannins, the dominant phenolic class, exhibit strong free-radical scavenging activity due to multiple hydroxyl groups capable of donating hydrogen atoms to neutralize reactive oxygen species (ROS). Moreover, ellagitannins chelate redox-active transition metals, such as iron and copper, inhibiting Fenton-type reactions that accelerate oxidative damage. By reducing intracellular ROS and limiting metal-catalyzed oxidation, these compounds help preserve collagen integrity and delay skin aging processes. Their anti-inflammatory activity, via downregulation of pro-inflammatory mediators, further contributes to improved skin tone, radiance, and resilience [13,14,15,16,17,18,19,20,21,22,23].

Vanillin, beyond its aromatic role, displays notable antioxidant and anti-inflammatory activity. In topical formulations, vanillin stabilizes lipophilic components, protecting them from oxidation, while simultaneously exerting mild protective effects on the skin surface through modulation of oxidative pathways. Its dual function enhances both sensory and protective properties of cork-derived ingredients [6].

Friedelin, a pentacyclic triterpenoid characteristic of cork, contributes additional bioactivity through anti-inflammatory and wound-healing effects. It promotes tissue repair by stimulating fibroblast activity and supporting extracellular matrix remodeling, making it particularly valuable in formulations aimed at barrier restoration and anti-aging care [9,24,25,26,27,28].

Complementary phenolic acids, such as gallic acid and caffeic acid, reinforce the antioxidant profile. Gallic acid supports an even skin tone by inhibiting melanogenesis-related oxidative reactions and strengthening the dermal barrier through radical-scavenging activity. Caffeic acid reduces inflammation, enhances microcirculation, and promotes tissue regeneration, crucial for maintaining healthy and rejuvenated skin. Collectively, ellagitannins, vanillin, friedelin, gallic acid, and caffeic acid exhibit synergistic activity across oxidative, inflammatory, and regenerative pathways, providing a robust multifunctional profile suitable for advanced topical formulations [13,14,15,16,17,18,19,20,21,22,23,29].

### 4.2. Anti-Aging Activity

Cork-derived bioactives exhibit anti-aging effects primarily through modulation of oxidative stress and preservation of cellular homeostasis in skin cells. Skin aging is strongly linked to excessive ROS production, which induces lipid peroxidation, protein oxidation, and DNA damage, ultimately compromising dermal structure and elasticity [30,31]. The phenolic fraction of cork extracts, enriched in flavonols, gallic acid, and caffeic acid, provides potent antioxidant and anti-inflammatory protection against these oxidative insults [9,32,33,34].

Flavonols act as multifunctional antioxidants by donating hydrogen atoms, chelating transition metals, and interrupting ROS chain reactions. Additionally, they upregulate endogenous antioxidant defenses, including superoxide dismutase (SOD), catalase (CAT), and glutathione peroxidase (GPx), while inhibiting pro-oxidant enzymes such as xanthine oxidase [35,36,37].

Gallic acid complements these effects by neutralizing hydroxyl and peroxyl radicals, protecting structural proteins from glycoxidation, and preventing UV-induced degradation. In human dermal fibroblasts and murine models, gallic acid reduces matrix metalloproteinase-1 (MMP-1) expression, limits collagen breakdown, and enhances dermal elasticity following UVB exposure [38,39]. Caffeic acid supports dermoprotection by enhancing intracellular glutathione levels and attenuating inflammatory cascades via NF-κB inhibition and Nrf2 pathway activation [40,41].

**Figure 5:**
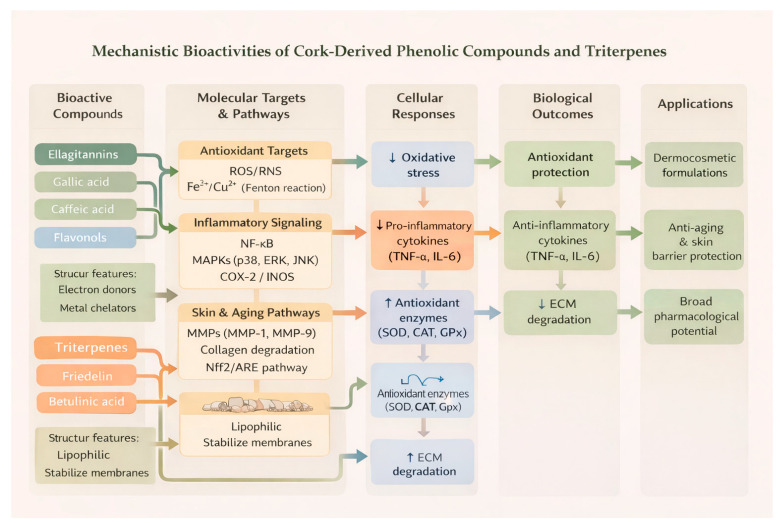
Overview of cork-derived bioactive compounds and their reported pharmacological potential.

Together, these mechanisms safeguard fibroblasts and keratinocytes from oxidative injury, maintain collagen integrity, and suppress inflammatory signaling associated with photoaging. The synergistic presence of flavonols, gallic acid, and caffeic acid underpins the multifunctional anti-aging activity of cork extracts, positioning them as promising dermocosmetic agents [9,10,32,33,42], Figure 5. Optimization of extraction and formulation strategies is recommended to enhance stability, bioavailability, and therapeutic efficacy.

### 4.3. Applications in Topical Formulations

Cork-derived bioactives, particularly phenolics and triterpenoids, have demonstrated potential for incorporation into advanced skincare formulations due to antioxidant, anti-inflammatory, photoprotective, and barrier-restoring properties [43,44,45]. These activities align with key therapeutic goals in dermocosmetics, where oxidative stress, inflammation, and impaired barrier function drive skin aging and dysfunction.

Anti-aging creams and serums: Cork extracts exert anti-aging effects through the synergistic action of flavonols, gallic acid, and caffeic acid. These compounds scavenge free radicals, modulate cellular antioxidant defenses, inhibit lipid peroxidation and protein oxidation, and preserve collagen and elastin fibers. Activation of the Nrf2 pathway and upregulation of antioxidant enzymes such as SOD and CAT further reduce ROS-induced cellular senescence, protecting dermal fibroblasts and supporting skin firmness and elasticity [43,44,45].

**Figure 6:**
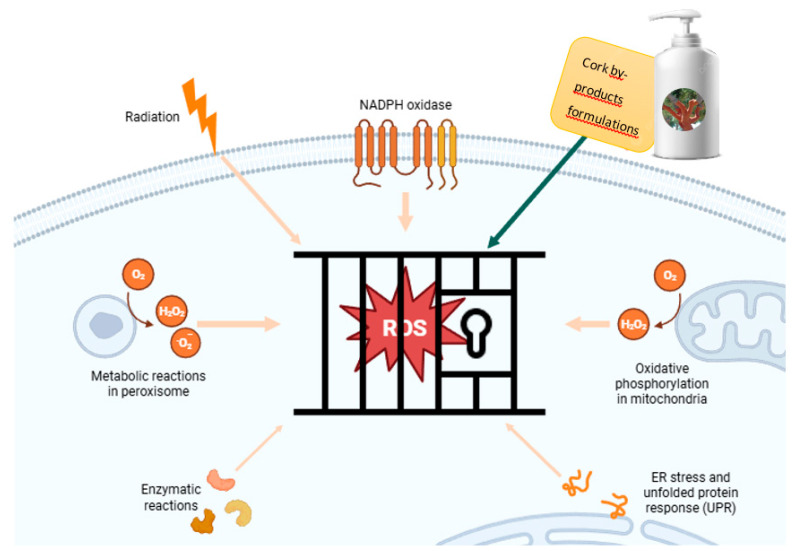
Protective mechanisms of cork-derived bioactives against oxidative stress in skin cells (*Created by Biorender*).

Sun-protective formulations: Cork phenolics enhance photoprotection by combining with conventional UV filters. Phenolic acids like caffeic and ferulic acid absorb UV radiation, dissipate photon energy, and inhibit UV-induced activation of MMPs and NF-κB signaling, reducing collagen degradation and inflammation [46,47]. Flavonoids have been shown to increase the sun protection factor (SPF) and improve photostability by neutralizing ROS generated during UV exposure [48] (Figure 6).

Moisturizers and barrier-repair products: Cork triterpenes such as betulinic acid and friedelin support skin barrier restoration and hydration by modulating keratinocyte lipid synthesis and upregulating filaggrin and involucrin expression [42,49]. Flavonols and related phenolics promote ceramide synthesis and enhance stratum corneum cohesion, reducing transepidermal water loss and improving cutaneous resilience [50].

Depigmenting and tone-evening products: Phenolic compounds, particularly gallic acid, inhibit tyrosinase and downregulate MITF and TRP-1 pathways, reducing melanogenesis and promoting uniform skin tone [49,51]. Cork-derived phenolics thus offer a multifunctional strategy for skin lightening by combining antioxidant, anti-inflammatory, and melanogenesis-modulating activities [49,51].

Collectively, cork bioactives demonstrate cosmeceutical versatility through complementary mechanisms that protect, repair, and rejuvenate the skin. Their natural origin, biocompatibility, and proven bioactivity support their integration into next-generation skincare products targeting oxidative stress, photodamage, barrier dysfunction, and pigmentary disorders.

## 5. Applications in Pharmaceutical Prototypes and Formulations

Cork-derived bioactive compounds, particularly phenolic acids, flavonoids, ellagitannins, and triterpenoids such as friedelin, betulinic acid, and lupeol, have been increasingly explored for their integration into pharmaceutical prototypes. Beyond their established role in dermocosmetics, these molecules possess pharmacological activities—including anti-inflammatory, antimicrobial, antioxidant, hepatoprotective, cardioprotective, neuroprotective, and anticancer effects—which provide a strong rationale for their therapeutic development [9,13,14,15,16,17,18,19,20,21,22,23,24,32,38,39,40,41].

### 5.1. Topical and Transdermal Formulations

The incorporation of cork extracts into topical pharmaceutical forms leverages both their antioxidant and anti-inflammatory properties to target skin disorders, wound healing, and photoaging. Formulations such as hydrogels, creams, and transdermal patches can improve bioavailability and stability of the active constituents while ensuring controlled release. The multifunctional activity of cork bioactives, including modulation of NF-κB and Nrf2 pathways, supports their use in formulations designed to reduce oxidative stress, inflammation, and tissue damage [43,44,45,46,47,48].

### 5.2. Oral and Systemic Delivery

Emerging studies indicate that cork-derived compounds can be incorporated into oral dosage forms, including capsules, tablets, and functional nutraceuticals. For instance, ellagitannins and gallic acid demonstrate significant bioavailability and systemic antioxidant activity, which can contribute to cardiovascular, hepatic, and neuroprotective effects [9,32,33,34,38,39,40,41]. Encapsulation techniques, such as liposomes or polymeric nanoparticles, have been proposed to enhance solubility, protect labile compounds from degradation, and enable targeted delivery.

### 5.3. Pharmaceutical Prototyping and Translational Perspectives

The progression from cork waste to bioactive ingredients allows the design of pharmaceutical prototypes that combine efficacy with sustainability. Standardization of extraction procedures, thorough chemical characterization, and biological validation are essential to ensure reproducibility and regulatory compliance. By integrating cork by-products into drug development pipelines, researchers can align with the principles of circular bioeconomy, valorizing industrial residues while creating high-value therapeutic agents [13,14,15,16,17,18,19,20,21,22,23,32].

### 5.4. Challenges and Future Directions

Despite the promising bioactivity profile, several challenges remain. The heterogeneity of cork residues, influenced by geographic origin, season, and tree age, can impact extract composition and bioactivity. Optimization of extraction, purification, and formulation protocols is necessary to achieve consistent quality and potency. Moreover, in vivo studies and clinical trials are required to confirm efficacy and safety in humans. Future research should focus on integrating cork-derived compounds into innovative drug delivery systems, exploring synergistic combinations, and expanding the therapeutic spectrum beyond topical applications [9,32,33,34,38,39,40,41].

Collectively, the translational potential of cork by-products exemplifies a sustainable approach to drug discovery, transforming previously undervalued residues into functional, high-value pharmaceutical ingredients.

## 6. Valorization of Cork By-Products

The sustainability profile of cork enhances its industrial appeal. Cork production is inherently eco-friendly, due to the regenerative nature of cork oak trees and the near-zero-waste practices employed during processing. Portugal, which accounts for the majority of global cork production, exemplifies a circular economy model in which industrial by-products are systematically valorized across sectors such as energy generation, agriculture, pharmaceuticals, and cosmetics. This intersection of environmental stewardship and functional innovation aligns with the growing societal demand for green solutions in health and well-being.

**Figure 7:**
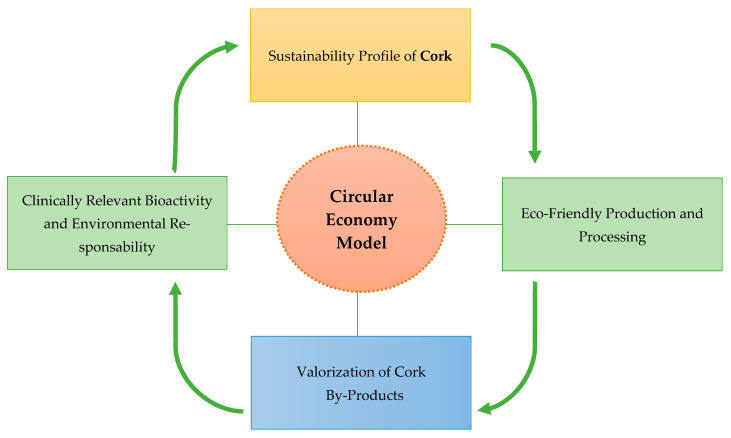
Valorization pathways of cork by-products within a circular bioeconomy framework.

As the cosmetic and pharmaceutical industries increasingly integrate natural bioactives with scientifically validated benefits, cork-derived compounds offer a dual advantage: clinically relevant bioactivity and environmental responsibility. Their incorporation into dermocosmetic and therapeutic formulations represents a compelling avenue for future development (Figure 7) [10,32,52,53,54,55,56,57,58,59,60,61].

The integration of cork by-products into pharmaceutical and cosmetic applications exemplifies the practical implementation of circular economy principles in bio-based industries. This approach not only maximizes the economic and environmental value of cork but also establishes a sustainable framework for the recovery and transformation of materials traditionally considered industrial residues. During cork processing for stoppers and construction materials, a substantial fraction of by-products—including cork powder, granulates, and lower-quality bark—remains underexploited despite its rich composition of bioactive secondary metabolites, particularly phenolic compounds, flavonoids, and triterpenoids. Through systematic valorization, these biologically active molecules can be harnessed as functional ingredients for cosmetic, dermatological, and pharmaceutical applications, effectively converting waste into high-value natural assets [10,32,57,58,62].

## 7. Future Perspectives and Sustainability Considerations

The valorization of cork by-products as bioactive ingredients aligns with the principles of green chemistry, circular bioeconomy, and sustainable pharmaceutical development. By transforming residues that were previously discarded into functional molecules, the cork industry can contribute to reducing waste, minimizing environmental impact, and creating new revenue streams.

### 7.1. Sustainable Extraction and Processing

Future research should prioritize eco-friendly extraction technologies, such as supercritical CO_2_, pressurized liquid extraction, or enzymatic-assisted processes, which minimize the use of toxic solvents and reduce energy consumption. Standardization of extraction conditions is essential to ensure reproducible yields of key bioactive compounds, including ellagitannins, phenolic acids, flavonoids, and triterpenoids [13,14,15,16,17,18,19,20,21,22,23,24,32]. Optimization of downstream processing and formulation can further enhance stability, bioavailability, and therapeutic efficacy while adhering to sustainable production practices.

### 7.2. Integration into Circular Bioeconomy

Cork by-products represent a renewable feedstock that can be valorized beyond pharmaceutical applications, including cosmetics, nutraceuticals, and functional foods. Developing integrated valorization strategies, from residue collection to high-value bioactive formulations, supports the circular bioeconomy by closing the loop in cork processing industries. Life cycle assessment (LCA) and techno-economic analyses can help identify the most sustainable pathways for industrial implementation [13,17,18,19,20,21,22,23,32].

### 7.3. Regulatory and Safety Considerations

For pharmaceutical and nutraceutical applications, comprehensive toxicological evaluation and compliance with regulatory frameworks are paramount. Studies should assess the potential cytotoxicity, allergenicity, and long-term safety of cork-derived extracts. Establishing quality control standards, including chemical fingerprinting and bioactivity assays, will facilitate regulatory approval and ensure consistent efficacy and safety in end-products [9,24,32,34,38,39,40,41].

### 7.4. Emerging Research Directions

Future investigations may explore synergistic combinations of cork bioactives with other natural compounds or conventional pharmaceuticals, aiming to enhance efficacy and broaden therapeutic potential. Advances in nanotechnology, encapsulation, and targeted delivery systems offer promising avenues for improving bioavailability and controlled release. Additionally, mechanistic studies focusing on molecular pathways—such as NF-κB, Nrf2/ARE, MAPKs, and COX/LOX—can provide deeper insight into pharmacological action and guide rational formulation design [13,14,15,16,17,38,39,40,41].

## 8. Conclusions

This review highlights the remarkable potential of *Quercus suber* L. (cork) as a sustainable and multifunctional raw material for pharmaceutical and cosmetic applications. The unique biochemical composition of cork—dominated by suberin, lignin, polysaccharides, waxes, and a diverse array of phenolic compounds—supports its role as a valuable source of bioactive molecules with clinically relevant properties. In particular, phenolic compounds are associated with strong antioxidant, anti-inflammatory, and anti-aging effects, supporting their integration into advanced topical and therapeutic formulations.

Building on this foundation, the biological activity of cork extracts has been repeatedly demonstrated in both in vitro and in vivo contexts. Of particular note is their ability to neutralize reactive oxygen species (ROS), inhibit oxidative enzymes, and modulate pro-inflammatory pathways. These mechanisms are directly linked to the prevention of skin aging, promotion of cellular resilience, and mitigation of inflammation-associated tissue degradation. Antioxidant activity assessed through FRAP, DPPH, and ABTS assays consistently shows that ethanolic cork extracts outperform aqueous ones, emphasizing the importance of solvent choice and temperature in maximizing extract efficacy.

However, despite the promising results observed thus far, further research is essential. Standardized methodologies for extraction, characterization, and formulation need to be developed to ensure consistency, reproducibility, and clinical efficacy. Moreover, controlled in vivo studies and clinical trials will be critical in translating laboratory evidence into real-world applications. Understanding the mechanisms of action at the cellular and molecular levels will also contribute to unlocking the full therapeutic potential of cork.

In summary, cork stands at the confluence of tradition, innovation, and sustainability. Its rich chemical profile, strong antioxidant and anti-inflammatory activity, and ecological credentials position it as a next-generation natural ingredient for the development of safe, effective, and environmentally conscious biomedical and cosmetic products. With continued scientific investment, cork may well emerge as a model raw material in the era of sustainable biotechnological innovation.

## Data Availability

No new data were created or analyzed in this study. Data sharing is not applicable to this article.

## Abbreviations

The following abbreviations are used in this manuscript:

R&D	Research and development
TPC	Total phenolic content
BHA	Butylated hydroxyanisole
BHT	Butylated hydroxytoluene
DPPH	2,2-diphenyl-1-picrylhydrazyl
ABTS	2,2′-azino-bis(3-ethylbenzothiazoline-6-sulfonic acid)
FRAP	Ferric Reducing Antioxidant Power
ROS	Reactive oxygen species
FC	Flavonoid content
